# Evaluation of the Infill Design on the Tensile Response of 3D Printed Polylactic Acid Polymer

**DOI:** 10.3390/ma14092195

**Published:** 2021-04-25

**Authors:** Tanner David Harpool, Ibrahim Mohammed Alarifi, Basheer A. Alshammari, Abdul Aabid, Muneer Baig, Rizwan Ahmed Malik, Ahmed Mohamed Sayed, Ramazan Asmatulu, Tarek Mohamed Ahmed Ali EL-Bagory

**Affiliations:** 1 Department of Mechanical Engineering, Wichita State University, 1845 Fairmount, Wichita, KS 67260, USA; ima804@gmail.com; 2 Department of Mechanical and Industrial Engineering, College of Engineering, Majmaah University, Al-Majmaah, Riyadh 11952, Saudi Arabia; i.alarifi@mu.edu.sa (I.M.A.); t.elbagory@mu.edu.sa (T.M.A.A.E.-B.); 3 Engineering and Applied Science Research Center, Majmaah University, Al-Majmaah, Riyadh 11952, Saudi Arabia; 4 Materials Science Research Institute, King Abdulaziz City for Science and Technology, Riyadh 11442, Saudi Arabia; bshammari@kacst.edu.sa; 5 Engineering Management Department, College of Engineering, Prince Sultan University, P.O. Box 66833, Riyadh 11586, Saudi Arabia; aaabid@psu.edu.sa (A.A.); mbaig@psu.edu.sa (M.B.); 6 Department of Metallurgy and Materials Engineering, University of Engineering and Technology, Taxila 47050, Pakistan; rizwanmalik48@yahoo.com; 7 Department of Civil and Environmental Engineering, College of Engineering, Majmaah University, Majmaah 11952, Saudi Arabia; a.sayed@mu.edu.sa; 8 Department of Civil Engineering, Faculty of Engineering Assiut University, Assiut 71518, Egypt; 9 Department of Mechanical Design, Faculty of Engineering Materia, Helwan University, Cairo 11724, Egypt

**Keywords:** 3D printing, infill shapes, finite element analysis, construct stress, strain diagrams, fused filament fabrication (FFF)

## Abstract

The current study explores the effects of geometrical shapes of the infills on the 3D printed polylactic acid (PLA) plastic on the tensile properties. For this purpose, by utilizing an accessible supply desktop printer, specimens of diamond, rectangular, and hexagonal infill patterns were produced using the fused filament fabrication (FFF) 3D printing technique. Additionally, solid samples were printed for comparison. The printed tensile test specimens were conducted at environmental temperature, Ta of 23 °C and crosshead speed, V_C.H_ of 5 mm/min. Mainly, this study focuses on investigating the percentage infill with respect to the cross-sectional area of the investigated samples. The mechanical properties, i.e., modulus of toughness, ultimate tensile stress, yield stress, and percent elongation, were explored for each sample having a different geometrical infill design. The test outcomes for each pattern were systematically compared. To further validate the experimental results, a computer simulation using finite element analysis was also performed and contrasted with the experimental tensile tests. The experimental results mainly suggested a brittle behavior for solidly infilled specimen, while rectangular, diamond, and hexagonal infill patterns showed ductile-like behavior (fine size and texture of infills). This brittleness may be due to the relatively higher infill density results that led to the high bonding adhesion of the printed layers, and the size and thickness effects of the solid substrate. It made the solidly infilled specimen structure denser and brittle. Among all structures, hexagon geometrical infill showed relative improvement in the mechanical properties (highest ultimate tensile stress and modulus values 1759.4 MPa and 57.74 MPa, respectively) compared with other geometrical infills. Therefore, the geometrical infill effects play an important role in selecting the suitable mechanical property’s values in industrial applications.

## 1. Introduction

An engineering design involves a basic idea, proper planning, material selection, and an optimized manufacturing process regardless of the desired product or component. The proper execution of all steps to make an engineering product or component results in significant benefits in terms of low expenditures, decrease in the chances of failures during service, and reduced manufacturing time, which gives significant advantages over other market competitors. Often, several possible options with different combinations are available, and it seems logical that there exists an optimal solution. A more state-of-the-art approach to address this complex issue is provided by the recent fast-growing field of 3D printing technology [1,2,3,4]. During the last decade, 3D printing has made significant developments in industrial products. One of the main advantages of 3D printing is converting industrial products from a proposal to an actual application in minimum time. Three-dimensional printing has a huge capacity to create any type of part. It allows the user to make various tailored inputs before the commencement of the printing procedure. A most valuable factor, (especially for structural parts) is the kind and ratio of component infill. It seems ideal to put an idea in the form of a digital file of any size and shape and bring it into the real world relatively quickly in the form of a solid entity by using 3D printing technology. However, some shortcomings include the lack of consistency in strength, quality, or reliability of the fabricated product that needs to be overcome for various infills. Lee JY et al. observed the various 3D printing industrial applications of innovative materials, such as ceramics, electronics, biomaterials, composites, and smart materials [4].

Fused filament fabrication (FFF) is an extensively adopted additive manufacturing technology for thermoplastics materials due to its user-friendliness, low cost, and rapid processing [5,6,7,8]. Nevertheless, elevated temperatures during this technique may result in potential thermal degradation, uneven corners and gaps, and low surface quality with shrinkage issues in the final product. Such issues with its relatively complex process due to a high number of input parameters make the optimization of this technique rather difficult [9,10]. Still, the FFF method is used to fabricate most polymers involving acrylonitrile butadiene styrene (ABS) [11,12,13,14], polylactic acid (PLA) [5,12,15,16,17,18,19], polyethylene terephthalate (PET), polycarbonate (PC), and so on. In 2019, Cuan-Urquizo et al. [15] and Harris et al. [16] comprehensively reviewed the relationship between materials, different process variables, and their corresponding impacts on the mechanical properties of FFF printed parts. The specific process included parameters/variables, and the material’s structure played a vital role in determining the final strength of printed parts. Furthermore, to obtain authentic and reliable estimation, a combination of characterization results should be considered. 

Wang et al. [17] investigated the relationship between the Izod impact strength of printed PLA and two printing variables, plate temperature and layer height, and found that printed PLA at higher plate temperature showed significantly higher Izod impact strength as compared to injection molded PLA. This enhancement was attributed to the induction of smaller PLA crystals by the fused layer modeling (FLM) method. Dong et al. [18] successfully prepared 3D printed flexural, impact, and tensile testing specimens of PLA and PLA/wood fiber composites by considering 3D printing parameters; infill density, layer height, and the number of shells. From this study, it was found that PLA specimen showed much higher mechanical properties as compared to composite samples. It was reported that closely bounded layer-by-layer cross-sectional PLA structures result in enhancement of mechanical properties. 

Additionally, weak interfacial bonding between PLA matrices and wood fibers (owing to randomly oriented fiber bundles of wood fibers) was the main cause of the relatively lower mechanical strengths of PLA/wood composites. Several researchers also investigated the effect of the process parameters on the flexural properties of the final product. The influences of raster angle, raster width, and layer height on the FFF-printed PLA flexural properties were investigated by Rajpurohit et al. [19]. The authors concluded that the layer height is the prime factor followed by the raster angle to enhance flexural strength. It was found that the flexural strength was inversely proportional to layer height and raster angle. They suggested that deposition of more layers at lower layer height with higher extrusion pressure (to deposit a thin layer) resulted in improved bonding of the layers and consequently, enhanced the flexural strength. It was also found that reduction in voids near the edge was affected by a variety of external perimeters and consequently increased the flexural strength [20]. Luzanin et al. [21] have studied the influence of raster angle, layer height, percentage infill, extrusion temperature, and speed on the flexural behavior of the PLA parts. They reported the highest flexural strength at 0° raster angle. Relatively higher flexural strength was found at a lower raster angle by Garg et al. [22]. Motaparti et al. [23] have investigated the influence of raster angle, build direction, and air gap on the flexural behavior of the fused deposition modelling (FDM) parts and reported higher flexural strength for the sample having conditions of 0°/90° raster angle and negative air gap. Rahman et al. [24] have systematically investigated the raster angle’s effect on the flexural, tensile, and compressive properties of FFF-produced polyether ether ketone (PEEK) materials. On the other hand, Bakarich et al. [25] explored the fiber-reinforced hydrogel composites fabricated by additive manufacturing 3D printing. A comprehensive review on metallic and ceramic parts produced by extrusion-based additive manufacturing was performed by Rane et al. [26]. To optimize the FFF process parameters for improving mechanical properties and the thermoplastic materials anisotropic behavior during their extrusion, numerous investigations have been carried out [10,13,14,26,27].

Angel et al. [13] explored the influence of tensile test sample geometry on anisotropic mechanical properties. They observed that the longitudinally printed specimens displayed ultimate tensile stress comparable to the vertically printed and transversal samples. Furthermore, the influence of the orientations of the PLA part builds on the tensile fatigue properties was explored by Afrose et al. [27]. The highest (38.7 MPa) ultimate tensile stress was found for the PLA specimen built-in X-direction compared to other samples. Despite all, certain differences are observed in the physical and mechanical properties of FFF and injection molding products. In this scenario, it is essential to evaluate the mechanical properties of the PLA 3D printed plastic parts processed by computer-based simulation and by the experimental technique (FFF). In the current work, four different shapes (solid specimen, rectangular, hexagonal, and diamond infill models) were used for 3D printed specimen according to ASTM D638-14 [28] standard to study the mechanical performance under the operating conditions. The tensile testing of printed tensile test specimens was carried out at room temperature, Ta of 23 °C and crosshead speed, V_C.H_ of 5 mm/min. Furthermore, to predict the performance of the printed specimens, finite element analysis (FEA) was also conducted for the experimental validations at the operating conditions. Unlike previously published studies, the current research aims to observe the infill structure effects on the tensile properties of 3D printed PLA components to provide the designers and industrial manufacturers with an understanding of the FFF of the 3D printing technique. The motivation of the present study is to introduce the best pattern configuration at constant percentage infill equal to 15% infill for different patterns of the tensile test specimen under the same operating conditions. On the other hand, the study presents a comparison between the infill percentages in solid infill configuration by means of 100% infill and other patterns with percentage of 15% infill.

## 2. Experimental Work

### 2.1. Materials

**Figure 1:**
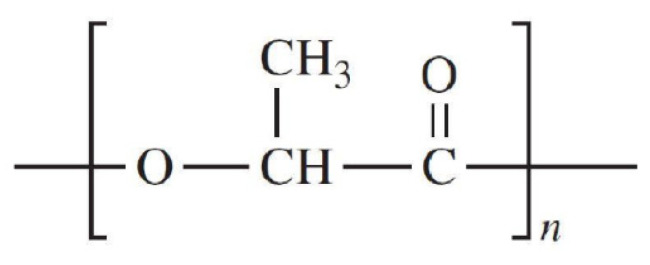
Chemical structure of PLA.

PLA filament was purchased from a commonly known online source (3DXTECH, Grand Rapids, MI, USA). PLA is a biodegradable “bioplastic” that is insoluble with water and is made from plant-based materials [29]. The chemical composition structure of PLA can be seen in Figure 1 [30]. Generally, PLA is obtained from tapioca root, cornstarch, and sugar cane and is widely recognized as an eco-friendly biodegradable 3D printing material. PLA commences to melt and soften at around 50 °C. Hence, a relatively lower temperature (180–190 °C) of the extruder nozzle is required to obtain a printable form of PLA [31,32].

### 2.2. Preparation of Infill Shapes for Tensile Test

**Figure 2:**
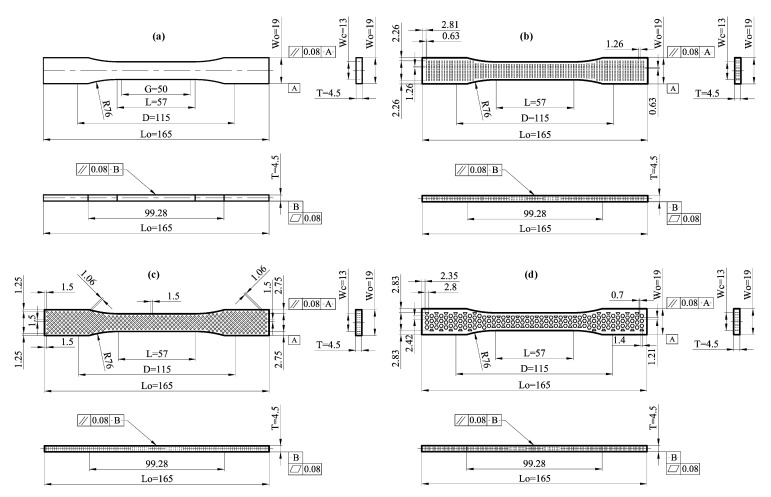
Dimensions of infill shapes for tension test specimen type I used for 3D printed specimen (**a**) solid, (**b**) rectangular, (**c**) diamond, and (**d**) hexagonal test specimen [20]. The infill percentage in rectangular, diamond, and hexagonal configuration is constant and equal to 15% infill. On the other hand, the infill percentage in the case of solid infill configuration is equal to 100% infill. All dimensions of tensile test specimen Type (I) are in mm according to ASTM D 638-14.

The thickness of the tensile test specimen has a significant effect during the selection of the specimen configuration and dimensions for the tensile test specimen. The standard tests have classified the configuration of tensile test specimen according to thicknesses of the applications, such as sheet, plate, pipe, and molded plastics. The main dimensions of geometrical infill for solid, rectangular, diamond, and hexagonal test specimens are identical geometrically. On the other hand, the difference between all configurations is based on printing geometry for the surface of the tensile test specimen. As per ASTM D638-14 [28] standard, Type I-dimension 3D printed samples were prepared with a total thickness, T, of 4.5 mm, a meter length or gauge length L, G, of 50 mm, and the grips gap, D, of 115 mm for all specimen configurations as shown in Figure 2a–d. In the tensile specimen with rectangular configuration, the width and height for rectangular geometrical infill are 0.63 mm and 1.26 mm, respectively, as shown in Figure 2b. The vertical and horizontal gap distance between each rectangular geometry and the other is about 0.63 mm and 1.26 mm, respectively. The vertical and horizontal gap distance between the rectangular geometry and test specimen outline is around 1.26 mm and 2.81 mm, respectively, as depicted in Figure 2b.

Figure 2c illustrates the specimen configuration and main dimensions from the type of diamond geometrical infill. The diamond geometrical infill is more different in the dimensions and orientations compared with rectangular geometry. The configuration of the diamond geometry is similar to the square configuration but with an orientation angle equal to 90° to configure the diamond geometrical infill by a square length equal to 1.06 mm. Furthermore, the diagonal length for diamond geometrical infill is equal to about 1.5 mm, while the gap distance between each diamond geometry and the other is about 1.5 mm in both vertical and horizontal directions. On the other hand, the vertical and horizontal gap distance between the diamond geometry and test specimen outline is around 1.25 and1.5 mm, respectively, from the lift side. Otherwise, from the right side, the vertical and horizontal gap distance is equal to 2.75 and 1.5 mm, as depicted in Figure 2c. The geometry of tensile specimens with hexagonal geometrical infill is more complicated. The time consumed for printing the geometrical infill is higher than the printing time for other geometrical infill specimens. The maximum width and height of the hexagonal geometry are 2.85 and 2.42 mm, respectively. Furthermore, the vertical and horizontal gap distance between the hexagonal geometry and test specimen outline is around 2.83 and 2.35 mm, respectively, from the lift side shown in Figure 2d.

### 2.3. 3D Print Preparation

**Figure 3:**
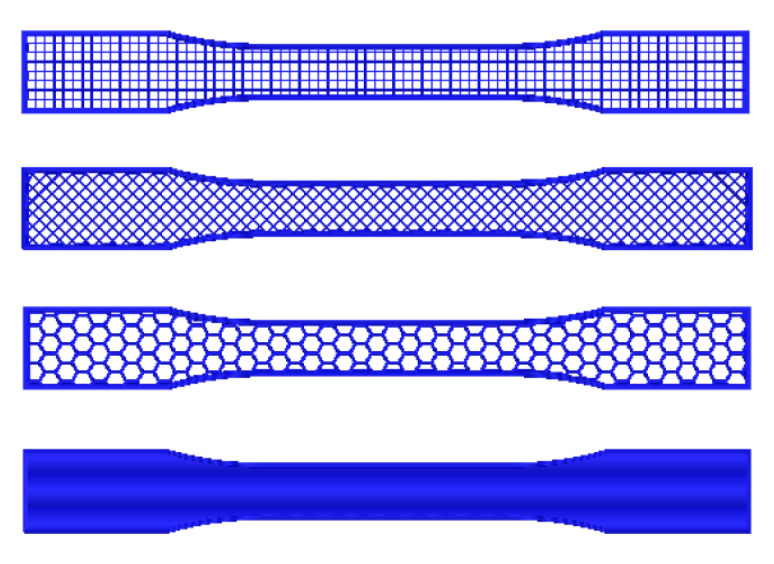
Various infills of Repetier-Host’s 3D previewer from top to bottom: rectangular, diamond, honeycomb (15% infill), and solid (100% infill).

**Figure 4:**
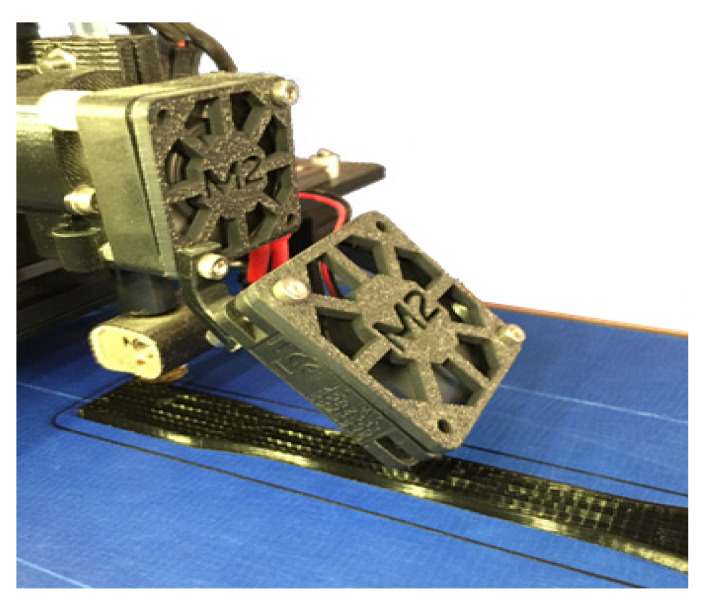
Picture of the printing process for rectangular shape infilled for the tensile test specimen. (Extruder nozzle temperature is equal to 215 °C and layer thickness is 0.3 mm.)

Filament feeding is the very first step in the printing procedure. This work was done by warming up the extruder and gradually supplying the filament. Then, Repetier-Host’s manual commands (Repetier Host 1.6.2, Hot-World GmbH & Co, Willich, Germany) were used for the extrusion of filament to make a certain smooth flowing and feeding. Then, by using the Repetier-Host interface, the print command was given. Three specimens for each geometrical infill shape (solid, rectangular, diamond, and hexagonal infill patterns presented in Figure 3) were printed for characterization. The infill design had connected each other through the same filament fibrils. Figure 4 shows an image of the printing of the rectangular shape infilled specimen. Universal (MTS) Test Works 4 software [33] was used in this study to record the load vs. elongation data, which were further processed to obtain stress vs. strain graphs. The printing parameters are printing temperature, (extruder nozzle temperature) is equal to 215 °C and layer thickness is 0.3 mm. The amounts of filament needed in linear millimeters are 1371, 1435, 1621, and 5016 mm for rectangular, diamond, honeycomb, and solid configuration, respectively. On the other hand, the estimated printing times are 9.54, 10.01, 11.12, and 27.58 min for rectangular, diamond, honeycomb, and solid configuration, respectively.

### 2.4. Finite Element Analysis

Modeling of PLA sheets was done using SOLID186 elements ANSYS-15 [34] software. The SOLID186 is a superior demand 3D 20-node solid component that displays quadratic displacement behavior. These elements support hyperelasticity, plasticity, stress stiffening, creep, huge strain, and deflection abilities. It can integrate design to simulate deformations of virtually incompressible elastic-plastic and completely incompressible hyperplastic materials. The PLA layers (integrated into the FE numerical simulation models) were supposed to develop linear elasticity. The stress–strain engineering and elastic modulus relationship, which is dependent on the experimental test samples and the Poisson ratio = 0.36, was used [35,36,37,38,39]. Tensile sample dimensions, various structures, and opening shapes were prepared according to a standardized sample PLA sheet [30], as represented in Figure 2. The meshing elements’ maximum size was 1 × 1 × 1 mm.

## 3. Results and Discussion

### 3.1. Tensile Testing Analysis

The analysis of tensile testing of printed polylactic acid material is carried out for all configurations at the same loading conditions with constant net cross-section area, A_o_. The net cross-section area at midsection for tensile test specimen is 13 × 4.5 mm according to ASTM D638-14 [28]. The engineering stress, σ, is calculated based on the applied force, P, dividing on the nest cross-section area A_o_. On the other hand, engineering strain, ε, is calculated by dividing the specimen elongation, ∆L, on the original gauge length, Lo. The modulus of the toughness of printed polylactic acid material is calculated by integrating the area under the engineering stress–strain curve according to the trapezoidal method for all configurations. The universal tensile testing machine that is used to conduct tensile tests for all specimens is MTS Criterion Model 45. The machine is equipped with the MTS Test Works 4 software and is capable of measuring and relaying the data to a computer. In order to increase the accuracy of output data, some of the important parameters of the tensile test are adjusted to ensure the test is aligned with the guidelines dictated in ASTM D638. The test speed is equal to 5.0 mm/min (initial speed was set to 2.0 mm/min) and a data acquisition rate is 5.0 Hz to ensure a proper number of data points.

#### 3.1.1. Solid Test Specimen

**Figure 5:**
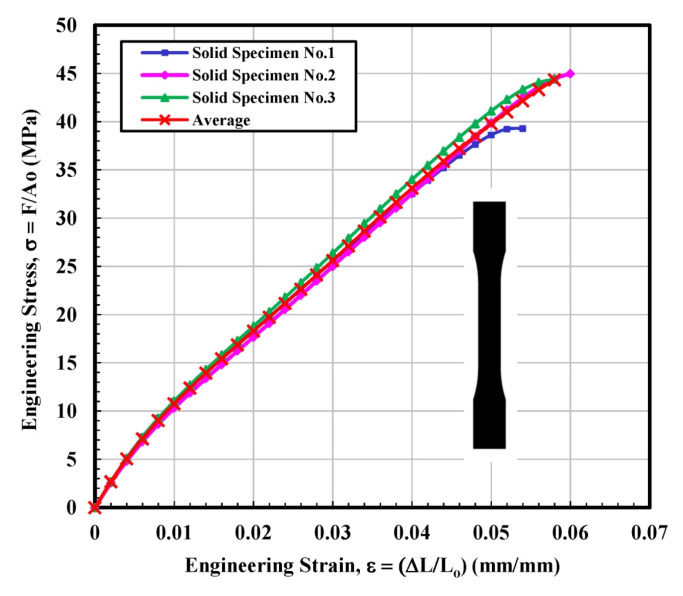
Engineering stress–strain curves for solid tested specimens.

To observe the behavior of the part up to a certain stress point or to examine the limits of a sample by bringing it to final breakdown, tensile tests were performed for all four infill-shaped 3D printed specimens. Figure 5 shows the stress–strain curves for the solid tensile tested specimen. This infill printed parts stress–strain relationship mainly exhibited elastic region before fracture and the behavior as a brittle material as a brittle material. The major portion of the stress–strain graph was almost linear, as shown in Figure 5. For solidly printed 3D specimens, it can be concluded that the stress–strain curves are very close to obeying Hook’s law (load vs. elongation are directly proportional) before fracture. The average calculated value of ultimate tensile stress (UTS) and modulus of elasticity were 44.34 MPa and 1275.5 MPa, respectively, as shown in Table 1. The fitting curve for average results of solid tested specimens is selected according to polynomial regression (degree = 8) and correlation coefficient, r within 0.996 to 0.999. The yield strength (44.97 MPa) is determined according to standard ASTM D638-14 [28].

#### 3.1.2. Rectangular Test Specimen

**Figure 6:**
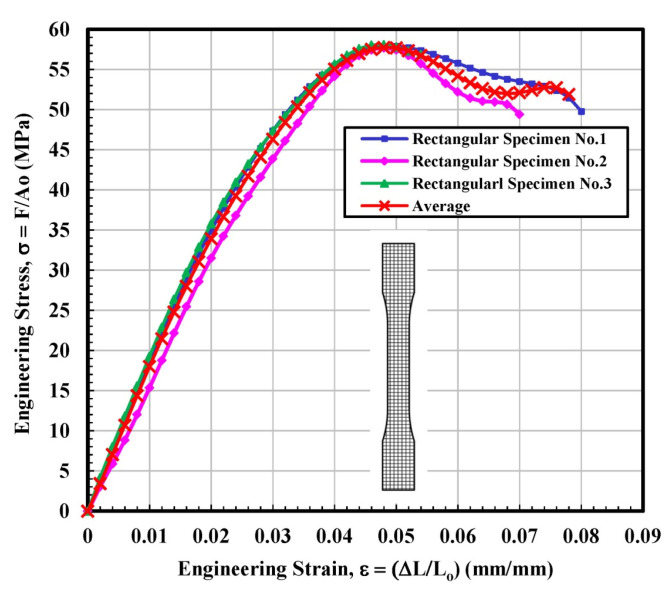
Engineering stress–strain curves for rectangular infill tested specimens.

Figure 6 observes the stress–strain curves for the rectangular tested specimen. The stress–strain lines of 3D printed parts with a rectangular shape displayed behavior closely resembling a ductile material. It seems very interesting as the filament (PLA plastic filament) used for all types of infill configurations, i.e., solid, rectangular, diamond, and hexagonal was the same. The main difference between solid and rectangular infills was the effective “loss” in the cross-sectional area. The difference in the stress vs. strain behavior suggests that the geometry of the 3D printed specimen significantly affects the mechanical properties. From Figure 6, nearly identical data were observed for the specimen 1 and 3 up to the ultimate tensile stress point. Soon after, the test sample 3 failed, while test sample 1 was able to withstand much longer. On the contrary, test sample 2 displayed slightly inconsistent behavior with a somewhat fluctuating (less smooth) curve. The wavering behavior of this sample may be attributed to the slight movement of the fibers or may be related to the simple resistance to the load in a different manner. The average calculated values of modulus of elasticity and ultimate tensile stress (UTS) were 1759.4 MPa and 57.74 MPa, respectively, which is higher than the average calculated values of solid infill tested specimen. The calculations of the fitting curve for the average results are selected according to polynomial regression (degree = 8) and correlation coefficient, r within 0.996 to 0.999. The maximum yield strength (44.97 MPa) is determined according to standard ASTM D638-14 [28], as shown in Table 2.

#### 3.1.3. Diamond Test Specimen

**Figure 7:**
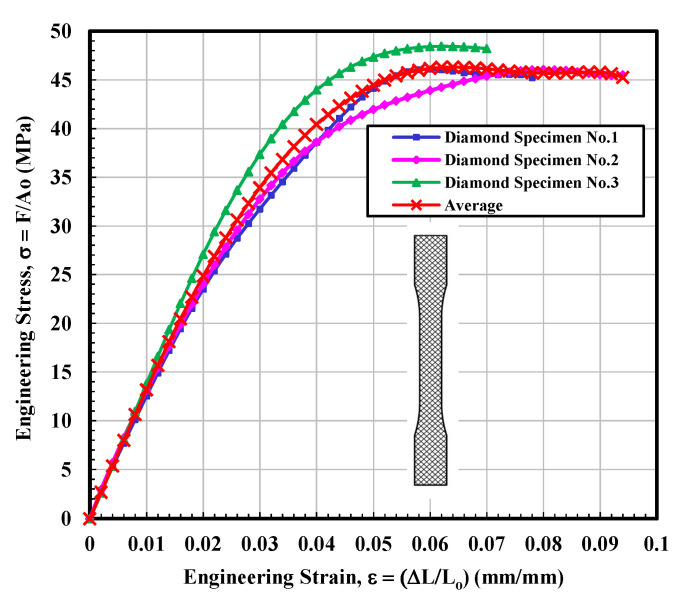
Engineering stress–strain curves for diamond infill test specimens.

Stress–strain curves for the diamond infilled samples are presented in Figure 7. A typical stress–strain diagram was seen for the diamond-infilled samples. It is observed that the obtained results for the three tested samples were somewhat inconsistent for different samples, and therefore, unique features were seen for each tested specimen. The stress vs. strain relationship observed for sample 1 exhibited very interesting phenomena around the point 0.025 mm/mm recorded strain, and a sudden drop in the measured stress was found. It is suggested that this sharp dip could result from the failure of fibers within the structures. This phenomenon is important for the mechanical performance of this specimen. It suggests that regardless of some structural damage (failure of fibers) around the point 0.025 mm/mm recorded strain, the sample still became able to reach a UTS value that is roughly comparable to the other samples’ UTS values. Tested sample 3 results were relatively normal as a function of applied stress until the maximum stress value, and then showed the almost constant value of stress until fracture, as can be observed from Figure 7. The average calculated values of modulus of elasticity and ultimate tensile stress (UTS) were 1300 MPa and 46.32 MPa, respectively, which is higher than the average calculated values of solid infill tested specimen but lower than that of average calculated values for 3D parts printed with a rectangular configuration as demonstrated in Table 3. Furthermore, the fitting curve for average results is selected according to polynomial regression (degree = 8) and correlation coefficient, r within 0.996 to 0.999. The yield strength is determined according to standard ASTM D638-14 [28].

#### 3.1.4. Hexagonal Test Specimen

**Figure 8:**
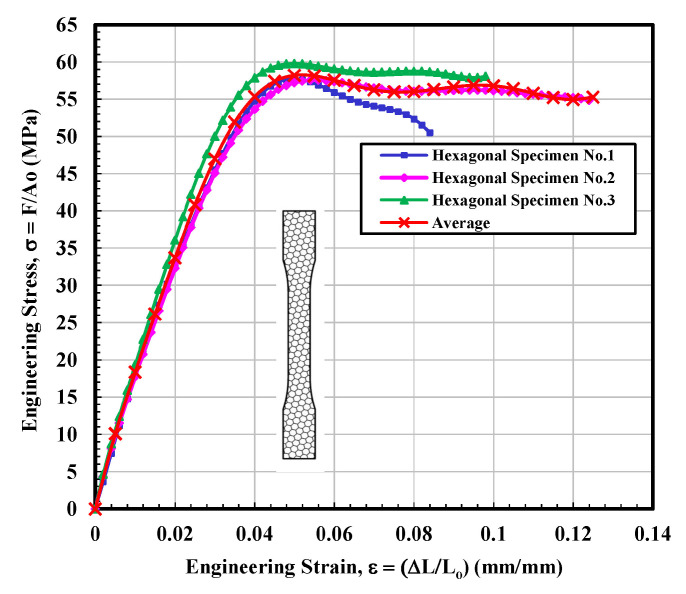
Engineering stress–strain curves for hexagonal infill test specimens.

**Figure 9:**
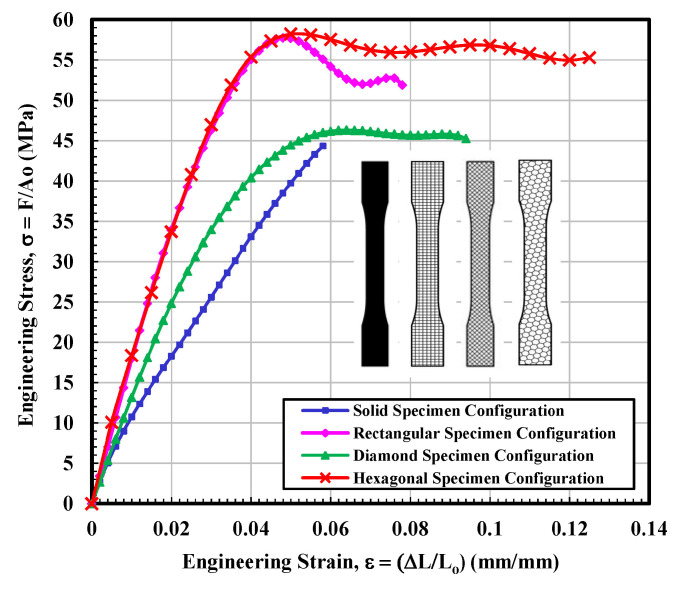
Average engineering stress–strain curves for solid, rectangular, diamond, and hexagonal infill test specimen configurations based on three tensile test specimens.

The Stress–strain curves for the hexagonal infill patterns are depicted in Figure 8. On the other hand, the behavior of curves seems a combination of the other three types. In terms of mechanical performance, especially modulus and UTS, the hexagonal infill unambiguously outperformed the 3D parts printed specimens with solid, rectangular, and diamond configurations, as can be observed from Figure 8. The general structure of the diagram and particularly modulus values showed very close resemblance to the 3D parts printed specimens with rectangular samples. Similarities were also found with the diamond-shaped infill specimen in terms of the resistance to failure. It can be noted from Figure 8 that the tested sample 2 exhibited a sharp decrease in stress value at around 0.07 mm/mm strain that seems quite similar to the stress–strain curve of diamond shape tested sample 1. However, this sharp dip was recorded after the UTS point. Table 4 illustrates that the average calculated values of modulus of elasticity and ultimate tensile stress (UTS) were 2016.8 MPa and 58.25 MPa, respectively, which is higher than the average calculated values of all infill tested specimens. On the other hand, the fitting curve for average results is selected according to polynomial regression (degree = 8) and correlation coefficient, r within 0.996 to 0.999. The yield strength is determined according to standard ASTM D638-14 [28]. The average calculated stress vs. strain curves for solid, rectangular, diamond, and hexagonal infill test specimen configurations based on three tensile test specimens are presented in Figure 9.

**Figure 10:**
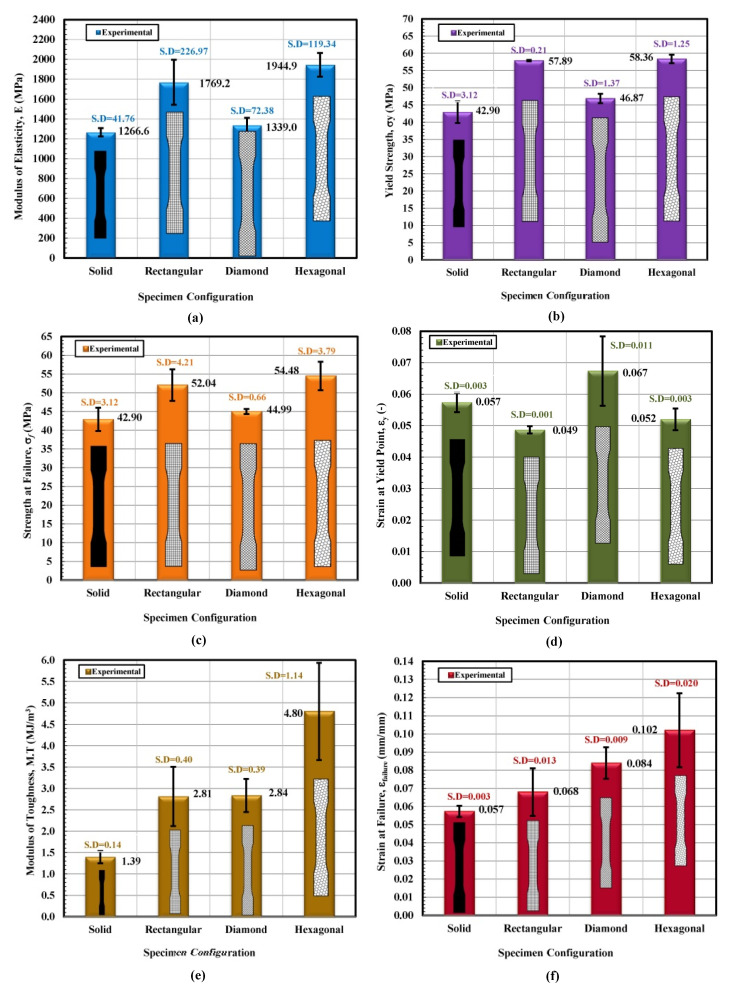
Stress vs. strain diagram showing an average trend line for each infill configuration as a function of (**a**) modulus, (**b**) yield strength, (**c**) strength at failure, (**d**) Strain at yield, (**e**) modulus of toughness, M.T, and (**f**) strain at failure.

A comparison of the tensile properties of various infill shapes was made and is presented in Figure 10a–f. It can be viewed from Figure 10a that the rectangular and hexagon infilled samples produced consistently higher modulus values. The hexagon infill exhibited the highest value compared to the other three infill configurations and the value was approximately 64% greater than a solid sample. However, the reported modulus value of “generic PLA” is about 2780 MPa by using conventional testing injection molding [37]. Established by these explanations, it can be determined that the PLA produced by using the 3D printed technique is more elastic than that produced by more conventional techniques. This will drastically improve the fracture toughness values of the 3D printed infills. Generally, a very good entanglement of polymer chains was observed for injection molding, due to high pressure that results in strong and stiff samples. Furthermore, during the mold filling process, asymmetrical flow is maintained inside the barrel due to elevated temperature that helps to increase the modulus of the specimen. On the contrary, the bottom and top filaments usually do not perfectly attach and bond with each other in the FFF process and consequently, these results in relatively porous structures with large gaps/voids between the strands [38].

In terms of yield stress, all three infill configurations yielded higher values as compared to the solid sample, and the largest value was found to be for the hexagonal infilled pattern. Its average value was found to be around 220% greater than the solid specimen (see Figure 10b). The diamond shape showed the highest value in terms of the total percent elongation, which was 56% greater than the solid specimen and even about 8% higher than the reported value [40]. The average percentage elongation of the rectangular specimen was 2.9%, slightly over half the calculated value for the diamond infill sample. It can be observed from Figure 10d that the rectangular-shaped infill specimen showed a greater average value of the percentage elongation at yield. Based on all results, it was found that the diamond-modeled 3D printed components revealed additional elastic behavior, and these samples started yielding at a relatively smaller value of stress. Hence, these test specimens have shown a greater ability to elongate and resist breaking the greatest out of all infill configurations tested. The maximum area value observed at the stress–strain diagram represents the value of modulus of toughness (M.T). The pattern with hexagonal infill was superior in M.T. The M.T value of 4.8 MJ/m^3^ was recorded on the stress–strain diagram. This recorded value is relatively higher than the solid specimen tested by ~245%. The second closest M.T value was obtained for the diamond pattern, as shown in Figure 10e, f, which show a comparison of the strain at break properties of various infill shapes. It was expected that the diamond infill configuration would demonstrate superior strain value at collapses as compared to its counterparts (see Figure 10f) and the diamond infill configuration displayed relatively greater resistance before eventual fracture, specifically observed for test 3. However, a large margin of error was noted. It is very interesting to note that the infill configuration showed strain at collapses value ~110% more than the solid baseline. It was found that processing variables such as raster width and angle, air gaps infill orientation and density, the thickness of layer and shell wall [41,42,43,44,45,46], layer orientation and height, the temperature of extruder and plate [15,43] significantly affect the mechanical performance of the finished product. It was proposed that the air gaps and shell values at 0 and 1 mm, respectively, resulted in improved mechanical properties [15].

Furthermore, very recently, it was detected that various line pattern combinations and 100% density of the infill reduced the difference between the printed lines and resulted in an enhancement in adhesion of the bottom and top layers. Comparable to the PLA at standard temperature and strain for dimensional and material reasons, the 0-degree written PLA is more rigid and more similar to the original strain [47]. This gives a value in the direction of the fiber; in general, the tensile and compressive properties are most excellent in this area. Thus, the tensile modulus and modulus have the lowest power in the z-direction [48]. Another contributing factor to improved tensile and flexural strength is the increased printing pace, which means a higher print bed adhesion with the printer could move the previous layer more efficiently [49]. Because of this, the significant increase in compressive force, the final product deformed and absorbed the stress before showing significant increases in compression. 

### 3.2. Finite Element Analysis

**Figure 11:**
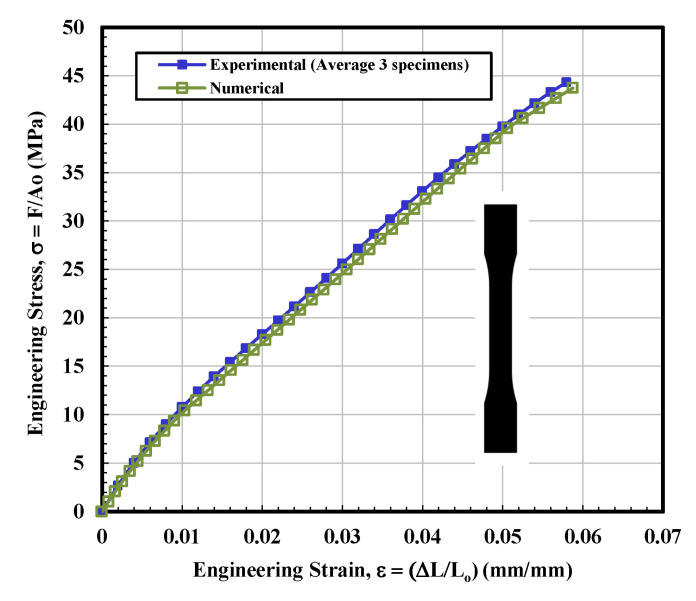
Engineering stress–strain curves for solid tested specimens based on experimental and finite element analysis.

**Figure 12:**
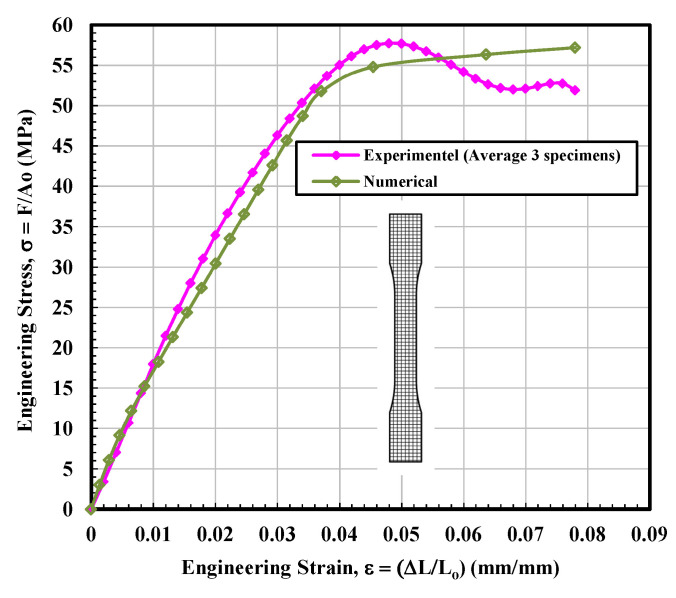
Engineering stress–strain curves for rectangular tested specimen based on experimental and finite element analysis.

**Figure 13:**
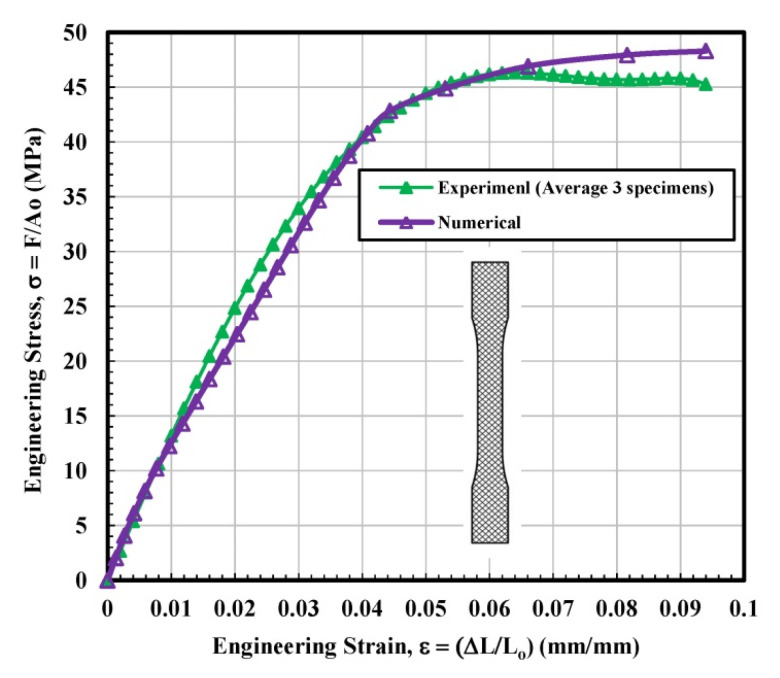
Engineering stress–strain curves for diamond tested specimens based on experimental and finite element analysis.

**Figure 14:**
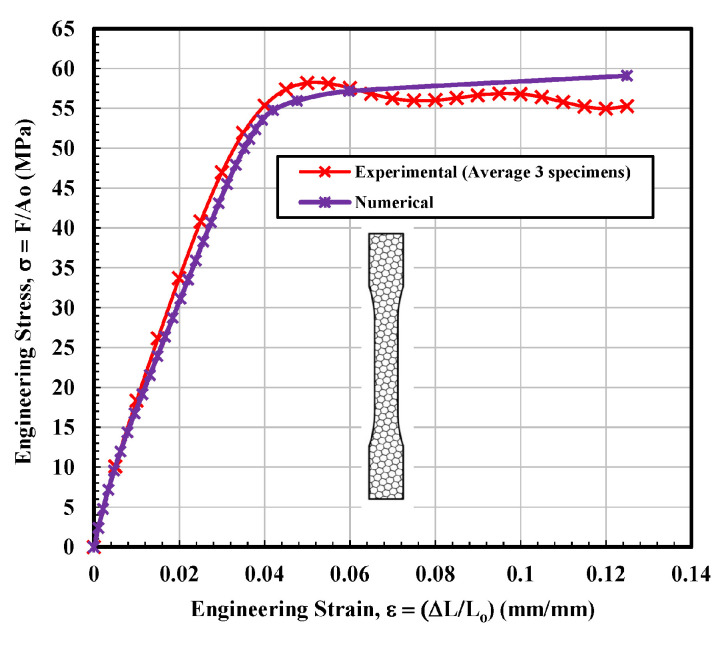
Engineering stress–strain curves for hexagonal tested specimen based on experimental and finite element analysis.

**Figure 15:**
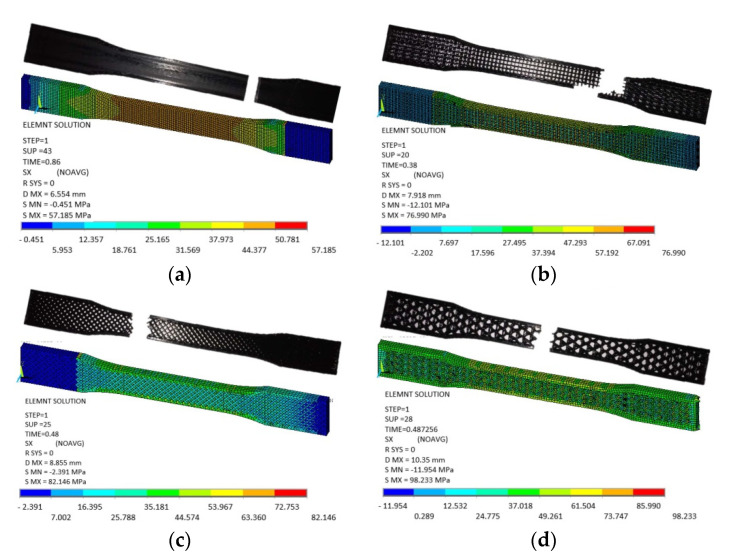
Stress distribution in the x-direction for (**a**) solid, (**b**) rectangular, (**c**) diamond, and (**d**) hexagonal infill test specimen configurations based on finite element analysis.

**Figure 16:**
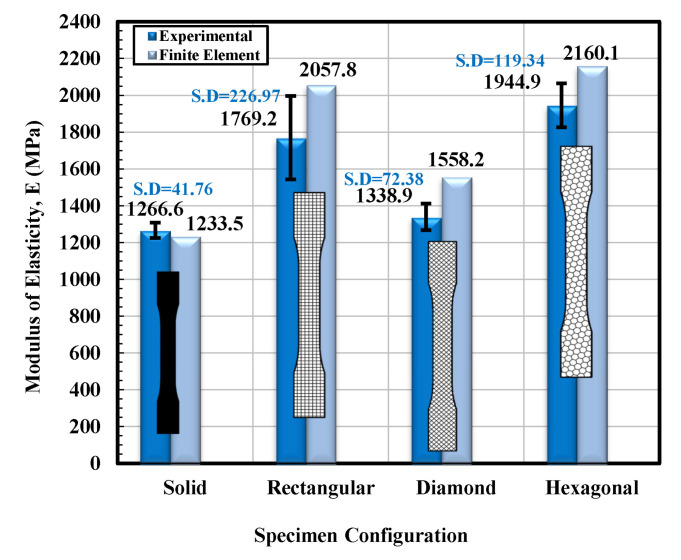
Apparent modulus of elasticity versus specimen configuration for both experimental and finite element analysis.

Based on the observations mentioned above, it is suggested that the behavior of solidly infilled specimen may be attributed to the relatively high infill density and good bonding adhesion between the printed layers that make the solidly infilled specimen structure stiffer and brittle, while rectangular, diamond, and hexagonal infill patterns showed ductile-like behavior, possibly due to relatively loose bonding between printed layers and fibril connections and structures. To further validate the obtained experimental results, an important software tool, finite element analysis, was applied, and experimental and simulated results were compared as shown in Figure 11, Figure 12, Figure 13 and Figure 14. These test results closely agreed with the experimental test results. Figure 15 show the stress distribution in the x-direction for solid, rectangular, diamond, and hexagonal infill test specimen configurations based on finite element analysis. Figure 16 represents the experimental and simulated outcomes of modulus of elasticity for various specimen configurations. It can be seen from Figure 16 that there is ~2–10% difference in experimental and simulated outcomes of both solid and hexagonal infill test specimens.

**Figure 17:**
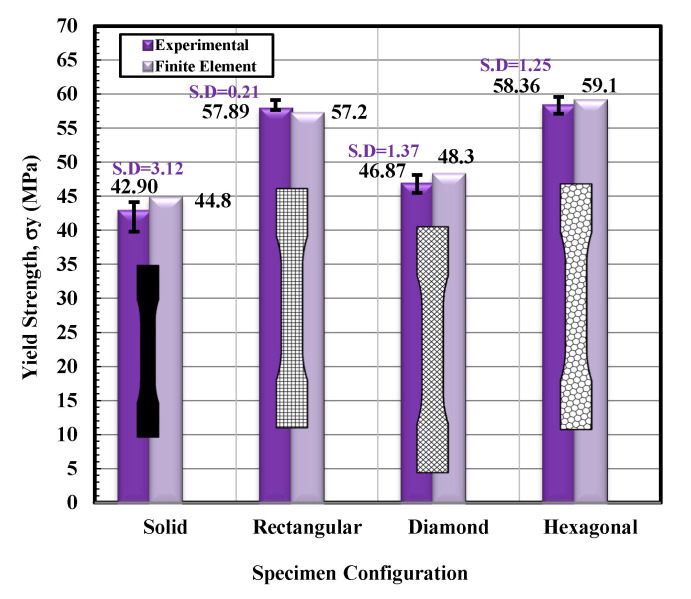
Yield strength versus specimen configuration for both experimental and finite element analysis.

**Figure 18:**
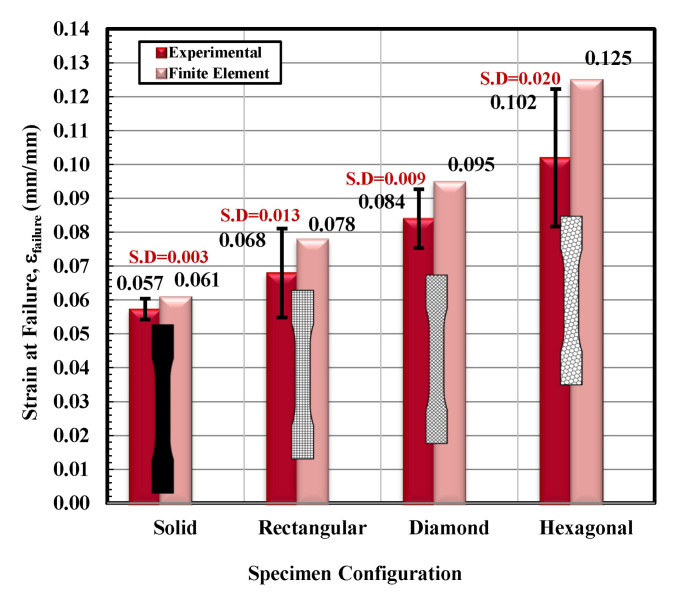
Strain at failure versus specimen configuration for both experimental and finite element analysis.

**Figure 19:**
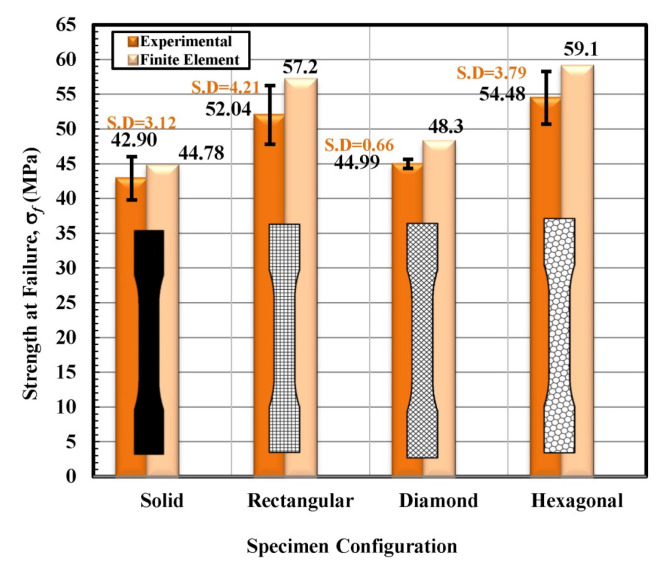
Strength at failure versus specimen configuration for both experimental and finite element analysis.

**Figure 20:**
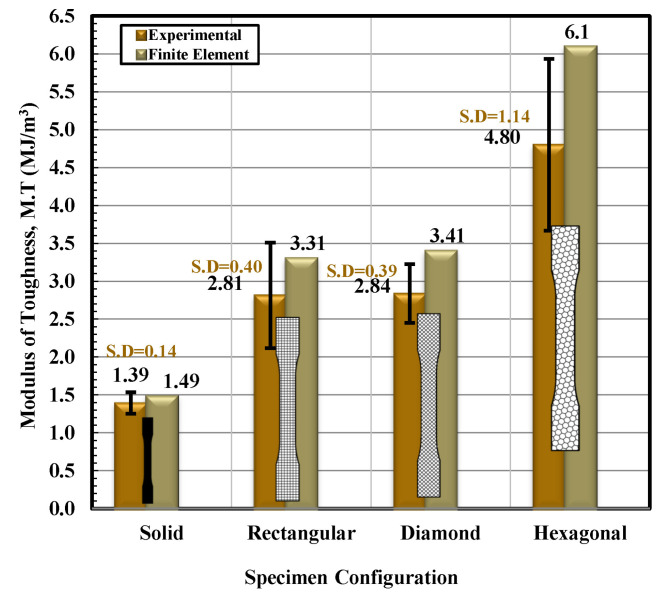
Modulus of toughness versus specimen configuration for both experimental and finite element analysis.

On the other hand, approximately ~14% difference in experimental and simulated results were found for the rectangular and diamond infill test specimen. The experimentally recorded and simulated yield strength results are shown in Figure 17 for solid, rectangular, diamond, and hexagonal infill configurations. In terms of yield strength, the difference between all recorded and simulated results was found to be less than ~2%. Similarly, there was a minute difference in finite element analysis and experimental results in terms of strain at failure (Figure 18), strength at failure (Figure 19), and modulus of toughness (Figure 20) for all specimen configurations. Based on the observations of finite element analysis and experimental results, it can be suggested that the attained outcomes from the tensile testing were accurate regardless of minor standard deviations.

## 4. Conclusions

Although infill is important for 3D printing, this research experimentally demonstrated that the structure and the infill fibers are similarly a very valuable factor of consideration. Furthermore, an FEA was applied to validate the experimental results. The obtained experimental and software-based results were systematically compared. Stress–strain curves showed brittle-like behavior for solidly infilled specimen with the lowest recorded modulus value (1287 MPa) and total percent elongation (2.5%). The failure occurred suddenly without any warning under the tensile loads. All non-solid specimens yielded relatively higher yield stress values, modulus, and ultimate tensile stress as compared to the solidly infilled specimen. Specifically, the hexagon configuration showed the highest modulus and ultimate tensile stress values with reasonable values of the other properties due to the structure strength. We observed ~2% difference in experimental and simulated outcomes of both solid and hexagonal infill test specimens. The pattern with hexagonal infill was found to be superior in terms of UTS. The UTS value of 58.5 MPa was recorded on the stress–strain diagram. This study is meaningful because the obtained data would provide comprehensive guidance to design various engineering parts for various industrial applications using different 3D printed plastic parts. It was found that the diamond-modeled 3D printed pattern has a higher yield strain than all sample patterns.

## Figures and Tables

**Table 1:** Experimental results of mechanical properties for solid tested specimens.

Mechanical Properties	Specimen No. 1	Specimen No. 2	Specimen No. 3	Average Value ± (Standard Deviation, S.D.)	Average Value According to Fitting Curve
E (MPa)	1291.9	1218.4	1289.5	1267 ± 42.0	1275.5
σ_y_ = σ_UTS_ (MPa)	39.31	44.97	44.41	43.0 ± 3.0	44.34
ε_y_ (mm/mm)	0.054	0.06	0.058	0.06 ± 0.003	0.058
σ*_f_* (MPa)	39.31	44.97	44.41	43.0 ± 3.0	44.34
ε_failure_ (mm/mm)	0.054	0.060	0.058	0.06 ± 0.003	0.058
M.T (MJ/m^3^)	1.23	1.49	1.46	1.0 ± 0.14	1.42

**Table 2:** Experimental results of mechanical properties for rectangular infill tested specimens.

Mechanical Properties	Specimen No. 1	Specimen No. 2	Specimen No. 3	Average Value ± (Standard Deviation, S.D.)	Average Value According to Fitting Curve
E (MPa)	1867.4	1509.7	1930.6	1769 ± 227	1759.4
σ_y_ = σ_UTS_ (MPa]	57.90	57.68	58.09	58.0 ± 0.21	57.74
ε_y_ (mm/mm)	0.05	0.048	0.048	0.05 ± 0.001	0.048
σ*_f_* (MPa)	49.80	49.42	56.90	52.0 ± 4.0	51.91
ε_failure_ (mm/mm)	0.08	0.07	0.054	0.07 ± 0.013	0.078
M.T (MJ/m^3^)	3.51	2.81	2.12	3.0 ± 0.70	3.34

**Table 3:** Experimental results of mechanical properties for diamond infill test specimen configuration.

Mechanical Properties	Specimen No. 1	Specimen No. 2	Specimen No. 3	Average Value ± (Standard Deviation, S.D.)	Average Value According to Fitting Curve
E (MPa)	1258.1	1397.7	1361.1	1339 ± 72	1300
σ_y_ = σ_UTS_ (MPa)	46.11	46.06	48.45	47 ± 1	46.32
ε_y_ (mm/mm)	0.06	0.08	0.062	0.07 ± 0.011	0.064
σ*_f_* (MPa)	44.25	45.49	45.24	45 ± 0.66	45.26
ε_failure_ (mm/mm)	0.08	0.094	0.078	0.080 ± 0.01	0.094
M.T (MJ/m^3^)	2.66	3.28	2.57	3.0 ± 0.40	3.0

**Table 4:** Experimental results of mechanical properties for hexagonal infill test specimen configuration.

Mechanical Properties	Specimen No. 1	Specimen No. 2	Specimen No. 3	Average Value ± (Standard Deviation, S.D.)	Average Value According to Fitting Curve
E (MPa)	1816.9	1964.7	2053.1	1945 ± 119	2016.8
σ_y_ = σ_UTS_ (MPa)	57.72	57.56	59.80	58 ± 1.0	58.25
ε_y_ (-)	0.05	0.056	0.05	0.050 ± 0.003	0.052
σ*_f_* (MPa)	50.52	54.84	58.08	54.0 ± 4.0	55.18
ε_failure_ (-)	0.084	0.124	0.098	0.10 ± 0.020	0.124
M.T (MJ/m^3^)	3.68	5.95	4.77	5.0 ± 1.0	6.03

## Data Availability

The data presented in this study are available on request from the corresponding author.

## References

[1] Harpool T.D. (2016). Observing the Effects of Infill Shapes on the Tensile Characteristics of 3D Printed Plastic Parts. Master’s Thesis.

[2] Page C., Kreuzer S., Ansari F., Eason D., Hamed E., Watson H. Optimizing 3D printed components: A methodological approach to assessing print parameters on tensile properties. Proceedings of the Annual Technical Conference—ANTEC, Conference Proceedings.

[3] Baich L. (2016). Impact of Infill Design on Mechanical Strength and Production Cost in Material Extrusion Based Additive Manufacturing. Master’s Thesis.

[4] Lee J.Y., An J., Chua C.K. (2017). Fundamentals and applications of 3D printing for novel materials. Appl. Mater. Today.

[5] Melnikova R., Ehrmann A., Finsterbusch K. 3D printing of textile-based structures by Fused Deposition Modelling (FDM) with different polymer materials. Proceedings of the IOP Conference Series: Materials Science and Engineering.

[6] Stansbury J.W., Idacavage M.J. (2016). 3D printing with polymers: Challenges among expanding options and opportunities. Dent. Mater..

[7] Mohamed O.A., Masood S.H., Bhowmik J.L. (2015). Optimization of fused deposition modeling process parameters: A review of current research and future prospects. Adv. Manuf..

[8] Padhi S.K., Sahu R.K., Mahapatra S.S., Das H.C., Sood A.K., Patro B., Mondal A.K. (2017). Optimization of fused deposition modeling process parameters using a fuzzy inference system coupled with Taguchi philosophy. Adv. Manuf..

[9] Alhnan M.A., Okwuosa T.C., Sadia M., Wan K.W., Ahmed W., Arafat B. (2016). Emergence of 3D Printed Dosage Forms: Opportunities and Challenges. Pharm. Res..

[10] Bhushan B., Caspers M. (2017). An overview of additive manufacturing (3D printing) for microfabrication. Microsyst. Technol..

[11] Rankouhi B., Javadpour S., Delfanian F., Letcher T. (2016). Failure Analysis and Mechanical Characterization of 3D Printed ABS with Respect to Layer Thickness and Orientation. J. Fail. Anal. Prev..

[12] Tymrak B.M., Kreiger M., Pearce J.M. (2014). Mechanical properties of components fabricated with open-source 3-D printers under realistic environmental conditions. Mater. Des..

[13] Torrado A.R., Roberson D.A. (2016). Failure Analysis and Anisotropy Evaluation of 3D-Printed Tensile Test Specimens of Different Geometries and Print Raster Patterns. J. Fail. Anal. Prev..

[14] Roberson D.A., Torrado Perez A.R., Shemelya C.M., Rivera A., MacDonald E., Wicker R.B. (2015). Comparison of stress concentrator fabrication for 3D printed polymeric izod impact test specimens. Addit. Manuf..

[15] Cuan-Urquizo E., Barocio E., Tejada-Ortigoza V., Pipes R.B., Rodriguez C.A., Roman-Flores A. (2019). Characterization of the mechanical properties of FFF structures and materials: A review on the experimental, computational and theoretical approaches. Materials.

[16] Harris M., Potgieter J., Archer R., Arif K.M. (2019). Effect of material and process specific factors on the strength of printed parts in fused filament fabrication: A review of recent developments. Materials.

[17] Wang L., Gramlich W.M., Gardner D.J. (2017). Improving the impact strength of Poly (lactic acid) (PLA) in fused layer modeling (FLM). Polymer.

[18] Dong Y., Milentis J., Pramanik A. (2018). Additive manufacturing of mechanical testing samples based on virgin poly (lactic acid) (PLA) and PLA/wood fibre composites. Adv. Manuf..

[19] Rajpurohit S.R., Dave H.K. (2018). Flexural strength of fused filament fabricated (FFF) PLA parts on an open-source 3D printer. Adv. Manuf..

[20] Mishra S.B., Malik R., Mahapatra S.S. (2017). Effect of External Perimeter on Flexural Strength of FDM Build Parts. Arab. J. Sci. Eng..

[21] Luzanin O., Guduric V., Ristic I., Muhic S. (2017). Investigating impact of five build parameters on the maximum flexural force in FDM specimens—A definitive screening design approach. Rapid Prototyp. J..

[22] Garg A., Bhattacharya A., Batish A. (2017). Failure investigation of fused deposition modelling parts fabricated at different raster angles under tensile and flexural loading. Proc. Inst. Mech. Eng. Part B J. Eng. Manuf..

[23] Motaparti K.P., Taylor G., Leu M.C., Chandrashekhara K., Castle J., Matlack M. (2017). Experimental investigation of effects of build parameters on flexural properties in fused deposition modelling parts. Virtual Phys. Prototyp..

[24] Rahman K.M., Letcher T., Reese R. Mechanical properties of additively manufactured peek components using fused filament fabrication. Proceedings of the ASME International Mechanical Engineering Congress and Exposition, Proceedings (IMECE).

[25] Bakarich S.E., Gorkin R., Panhuis M., Spinks G.M. (2014). Three-dimensional printing fiber reinforced hydrogel composites. ACS Appl. Mater. Interfaces.

[26] Rane K., Strano M. (2019). A comprehensive review of extrusion-based additive manufacturing processes for rapid production of metallic and ceramic parts. Adv. Manuf..

[27] Afrose M.F., Masood S.H., Iovenitti P., Nikzad M., Sbarski I. (2016). Effects of part build orientations on fatigue behaviour of FDM-processed PLA material. Prog. Addit. Manuf..

[28] ASTM International (2014). Standard Test Method for Tensile Properties of Plastics (Metric).

[29] Ghanbarzadeh B., Almasi H., Entezami A.A. (2010). Physical properties of edible modified starch/carboxymethyl cellulose films. Innov. Food Sci. Emerg. Technol..

[30] Harpool T.D., Alamir M., Asmatulu R. Effects of infill shapes on mechanical behaviors of 3D printed plastics. Proceedings of the CAMX 2017—Composites and Advanced Materials Expo.

[31] Giordano R.A., Wu B.M., Borland S.W., Cima L.G., Sachs E.M., Cima M.J. (1997). Mechanical properties of dense polylactic acid structures fabricated by three dimensional printing. J. Biomater. Sci. Polym. Ed..

[32] Costa S.F., Duarte F.M., Covas J.A. (2017). Estimation of filament temperature and adhesion development in fused deposition techniques. J. Mater. Process. Technol..

[33] (1995–2010). TestWorks 4.

[34] ANSYS (2015). User’s Manual.

[35] Menčík P., Přikryl R., Stehnová I., Melčová V., Kontárová S., Figalla S., Alexy P., Bočkaj J. (2018). Effect of selected commercial plasticizers on mechanical, thermal, and morphological properties of poly(3-hydroxybutyrate)/Poly(lactic acid)/plasticizer biodegradable blends for three-dimensional (3D) print. Materials.

[36] Farah S., Anderson D.G., Langer R. (2016). Physical and mechanical properties of PLA, and their functions in widespread applications—A comprehensive review. Adv. Drug Deliv. Rev..

[37] Anakhu P.I., Bolu C.C., Abioye A.A., Onyiagha G., Boyo H., Jolayemi K., Azeta J. (2018). Innovative foundry technology and material using fused deposition modeling and polylactic acid material in sand casing. Arch. Foundry Eng..

[38] Aloyaydi B., Sivasankaran S., Mustafa A. (2020). Investigation of infill-patterns on mechanical response of 3D printed poly-lactic-acid. Polym. Test..

[39] Shao J., Zhang Z., Zhao S., Wang S., Guo Z., Xie H., Hu Y. (2019). Self-Healing Hydrogel of Poly (Vinyl Alcohol)/Agarose with Robust Mechanical Property. Starch/Staerke.

[40] Polylactic Acid (PLA) Typical Properties (2016). Prospector. ULProspector, Web. https://plastics.ulprospector.com/generics/34/c/t/polylactic-acid-pla-properties-processing.

[41] Srinivasan R., Prathap P., Raj A., Kannan S.A., Deepak V. (2020). Influence of fused deposition modeling process parameters on the mechanical properties of PETG parts. Mater. Today Proc..

[42] Masood S.H., Mau K., Song W.Q. (2010). Tensile properties of processed FDM polycarbonate material. Materials Science Forum.

[43] Hossain M.S., Ramos J., Espalin D., Perez M., Wicker R. Improving tensile mechanical properties of FDM-manufactured specimens via modifying build parameters. Proceedings of the 24th International SFF Symposium—An Additive Manufacturing Conference, SFF 2013.

[44] Rodríguez-Panes A., Claver J., Camacho A.M. (2018). The influence of manufacturing parameters on the mechanical behaviour of PLA and ABS pieces manufactured by FDM: A comparative analysis. Materials.

[45] Goh G.D., Dikshit V., An J., Yeong W.Y. (2020). Process-structure-property of additively manufactured continuous carbon fiber reinforced thermoplastic: An investigation of mode I interlaminar fracture toughness. Mech. Adv. Mater. Struct..

[46] Luis E., Pan H.M., Sing S.L., Bastola A.K., Goh G.D., Goh G.L., Tan H.K.J., Bajpai R., Song J., Yeong W.Y. (2019). Silicone 3D Printing: Process Optimization, Product Biocompatibility, and Reliability of Silicone Meniscus Implants. 3D Print. Addit. Manuf..

[47] Bakis C.E., Haluza R.T., Bartolai J., Kim J.J., Simpson T.W. (2019). Assessment of anisotropic mechanical properties of a 3D printed carbon whisker reinforced composite. Adv. Compos. Mater..

[48] Goh G.D., Toh W., Yap Y.L., Ng T.Y., Yeong W.Y. (2021). Additively manufactured continuous carbon fiber-reinforced thermoplastic for topology optimized unmanned aerial vehicle structures. Compos. Part B Eng..

[49] Christiyan K.G.J., Chandrasekhar U., Venkateswarlu K. (2016). A study on the influence of process parameters on the Mechanical Properties of 3D printed ABS composite. IOP Conf. Ser. Mater. Sci. Eng..

